# Supplementary material for: Increased Motility in Campylobacter jejuni and Changes in Its Virulence, Fitness, and Morphology Following Protein Expression on Ribosomes with Altered RsmA Methylation
Source: Int J Mol Sci. 2024 Sep 10;25(18):9797. doi: 10.3390/ijms25189797 (PMC11431728; doi:10.3390/ijms25189797)
Supplement: Supplementary file 1 [file ijms-25-09797-s001.zip › suplementary materials.pdf]

International Journal of *Molecular Sciences*

MDPI

Supplementary Materials:

A

Protein	Gene	Abundance Ratio		Abundances		
		Δ*rsmA* / WT	Δ*rsmA* / Δ*rsmA*::*rsmA*	WT	Δ*rsmA*	Δ*rsmA* ::*rsmA*
PbpC	*cjj81176_0680*	0.9	2.7	121.1	110.6	40.7
Pgp2	*cjj81176_0915*	0.7	7.1	126	98.4	13.7
Cjj1105	*cjj81176_1105*	1.8	1.3	72.2	132.6	98.9

B

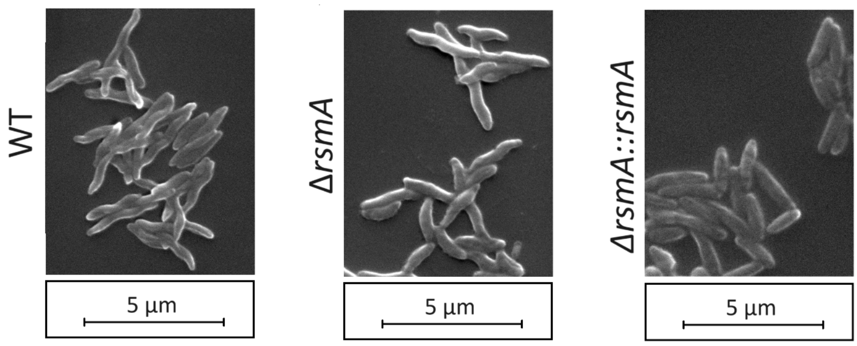

Figure S1. ***C. jejuni*** **cell morphology.** A) Significant changes in the expression of *C. jejuni* proteins that are associated with cell shape. The abundances of upregulated and downregulated proteins, compared with the wild-type strain, are marked in shades of red and blue, respectively. B) Representative electron micrographs showing the morphology of individual cells.

Table S1. Significant differences in the proteome of *C. jejuni* caused by inactivation of *rsmA*. Upregulated and downregulated proteins with significant changes of P > 0.05 are marked in red and blue, respectively.

Table S2. Nucleotide sequence of the 60-bp DNA fragment covering the start codon of genes encoding up- and down-regulated proteins. The regions corresponding to the translation start sites and Shine Dalgarno sequences are highlighted in red and yellow, respectively. The first three amino acids of the ORFs are shown.

Protein	UPREGULATED	N-terminal amino acids
**FlaG**	TCGATATAAGCTTTTAACTAGCAATAGGAAATTTTAAAAAGGATTTTAAAATGGAAATAT	Met-Asp-Ile
**FlgE**	AAAGCCAAAGCGTTAAATTTTTTAAAGCAATATTTTATAAAGGATTTAAGATGATGAGAT	Met-Met-Ser
**FlaB**	AACACTTCTTGCTTTAATCTTTTCGATGCAATATTTTGAAAGGATTTAAAATGGGTTTTA	Met-Gly-Phe
**FlaA**	GCATTTAACAAGCTCATGGATGAGTTTGAAATTATTTAAAAGGATTTAAAATGGGATTTC	Met-Gly-Phe
**FliD**	GAGTATTTTAGGGATGTGATAGGAATGATATTTGATAAGGAGAGTTAAAAATGGCATTTG	Met-Ala-Phe
**FlgG**	TAAGCTTGCAACTGTTAAATAATTTAAAATAAAATAAAAAAGGATTGAAAATGATGAGAT	Met-Met-Ser
**FlhA**	TATGAAAAATAATGGCAAAAAATAAAATAGTCGATTTAGTTTTTCCTTTCTTGGGGCCTT	Met-Gly-Pro
**CJJ0996**	ATTGAAATTTAGATATTTTGAAGTAGATAAGTAGAATTTAAAGGTTAGATATGGCAAAAA	Met-Ala-Lys
**FlgP**	GCTTATTGCTTTTTATTTTTTACTAATTACAATATTTTGAAGGTGTAAAAATGAAAAAAA	Met-Lys-Lys
**PseA**	AAAATTTAATTATAACGATATATATATTTAAGACTAATTAAAGGAAATTTATGAAATTTT	Met-Lys-Phe
**RpsL**	AATTTAGATATCATCCAAAGTTTATTACATTTTTAAAGAAAGGAATTATTGTGCCTACCA	Met-Pro-Thr
**HisF-1**	AAAGTCAAAATTTAGGTTTAAAACTTTTAGAAAATTTTGCAAGGCTTTAAATGTTAAAAA	Met-Leu-Lys
**HisH-1**	ATAAGAGATTATGATGGTTCTTTGGTAAGAAAAGACGAGTGTGTTTTAAAATGATAGCGC	Met-Ile-Ala
**PstS**	TTCCAAGTTTTTTTAAACTTCCATTACTTTTTTAAAGTTAAGGAGAAAAAATGAAAAAAA	Met-Lys-Lys
**Cjj1656**	TCACAAAAGATTGTGGAGTTTGTAAAGAACAAATTCCTATTTTACAGAATTTGGCTAAAA	Met-Ala-Lys
**PepP**	TCAAAAAACCTATCAATAAAATACTAAAAAAATTAATATTAAGGAGAAAATATGAGTATTT	Met-Ser-Ile
**Cjj1105**	TCTAGTTTTTGGTTCTTTTATGCTGGTAGAAAATTTTTTAAAGGAGTGGTGTGGTAAAAA	Met-Val-Lys
**Cpp33**	TTGAATTATCTTGTTGATAATCGCACACCAAAAGATCCAAAGAAGTTGCAATGAGTGATA	Met-Ser-Asp
**Ssb**	AGGAGTTTAAAAGATGAAAAAATATTACACCAACAAAAAGGAGTTAAACAATGAATAATG	Met-Asn-Asn
**Cpp35**	CAAATATAGAATCTGAAGAAACACAAGTATTTTAAGATTTAAGGATAAAAATGTCTATAA	Met-Ser-Ile
	**DOWNREGULATED**	
**RluB**	TATTTTTGTAATGTAAAAAAGGGCAAAAACGCCCTTTTAAGGAAGATCAAATGAGAATTA	Met-Arg-Ile
**GlnH**	ATAAGATAAAATTAGTATAATTTTTTTACAAAAAATTTTGAAAGGAAATTATGAAAAAAA	Met-Lys-Lys
**VirB8**	GTGCGTGCAATTATGATGTAATTAAAATTAGTTAAAACGAAAGGTAAAAAATGGCTTTTA	Met-Ala-Phe
**VirB9**	ATTGACAATGATACTTTTAACAATACACAACAAACGAAAGGAGCGAAGTAATGAGAAAAT	Met-Arg-Lys
**Cjp07**	GTTGTTTAGAGTATGAAAAATTATAGCCTAAATACAAAAAAGGAGCAATAATGGCAAGCA	Met-Ala-Ser
**Cjp08**	ATTGCAAAGCGTATAAATTAAGGACTACATATTTTAAAAAGGAAAAAATTATGGCAAGAA	Met-Ala-Arg
**VirB4**	TTGCAAAATTATCGATACCGCAAGACAAATATTATGCTTAAGGTTAAAAAATGGGAACGA	Met-Gly-Thr
**Cpp20**	AGATACTGCAAAAAAACAATAAAACAAAAATTTATCATAGGAGAATAAAAATGGAAAAAG	Met-Glu-Lys
**Cpp47**	AAAATGCAAAAAACAAAAACAAATCATAAAAAATTTAAAAAGGAGAAAAAATGGCATTTG	Met-Ala-Phe
	**CELL SHAPE PROTEINS**	
**PbpC**	TTTTTATAGAATGTTGTTTAGCTTATGTGTTTTTTAAAGGTAAAATTTAAATGAGAATGC	Met-Arg-Met
**Pgp2**	ATCACAAACATTTTTGACTTAAAAAATTTTGAAATACCTAAGGAAAAACATTGTTAAAAC	Met-Leu-Lys
